# Roles of Oral Infections in the Pathomechanism of Atherosclerosis

**DOI:** 10.3390/ijms19071978

**Published:** 2018-07-06

**Authors:** Ghazal Aarabi, Guido Heydecke, Udo Seedorf

**Affiliations:** Department of Prosthetic Dentistry, Center for Dental and Oral Medicine, University Medical Center Hamburg-Eppendorf, Martinistrasse 52, 20246 Hamburg, Germany; g.aarabi@uke.de (G.A.); g.heydecke@uke.de (G.H.)

**Keywords:** coronary artery disease risk, stroke risk, myocardial infarction, periodontitis, endodontic lesions, chronic inflammation

## Abstract

Oral infections occur frequently in humans and often lead to chronic inflammations affecting the teeth (i.e., caries), the gingival tissues surrounding the teeth (i.e., gingivitis and endodontic lesions), and the tooth-supporting structures (i.e., periodontitis). At least four basic pathogenic mechanisms have been proposed that involve oral inflammations in the pathogenesis of atherosclerosis: (1) low level bacteremia by which oral bacteria enter the blood stream and invade the arterial wall; (2) systemic inflammation induced by inflammatory mediators released from the sites of the oral inflammation into the blood stream; (3) autoimmunity to host proteins caused by the host immune response to specific components of oral pathogens; (4) pro-atherogenic effects resulting from specific bacterial toxins that are produced by oral pathogenic bacteria. In this narrative review, we summarize published experimental evidence related to these four mechanisms and discuss their impact on the pathogenesis of atherosclerosis.

## 1. Introduction

Atherosclerosis is a chronic inflammatory condition affecting arterial blood vessels [1,2]. Due to its causal role in the pathogenesis of cardiovascular diseases (CVD), atherosclerosis is closely related to one of the most frequent causes of morbidity and mortality worldwide [3]. In Europe, CVD is responsible for about 40% of all deaths, killing over 3.9 million people each year, the majority of deaths resulting from heart disease and stroke [4]. Coronary death, myocardial infarction (MI), and stroke represent “hard” or major CVD events, whereas examples for “soft” outcomes are angina pectoris, revascularization and peripheral artery disease, which are not so life threatening [1].

About 50% of MIs occur without significant previous symptoms and despite significant progress in the acute care of MI patients, the 4-week lethality after the first MI could not be lowered significantly below 50% [5]. Therefore, early detection of high-risk patients plays an important role in prevention. The classic risk factors identified i.e., on the basis of the Framingham Heart Study [6], account for only about half of the cardiovascular events occurring in the population, and almost half of all hard CVD events occur in patients lacking a classic risk factor [7,8]. For instance, the fraction of CHD deaths occurring in people with cholesterol levels that are lower than the population mean amounts to about 40% [6,7]. The precise causes underlying the “missing risk factor paradox” are currently unclear. One hypothesis is that the contribution of inflammation to CVD risk is not sufficiently covered by the existing risk models. Inflammation may come, for instance, from oral infections which occur locally in the oral cavity and which have the ability to modulate the course of atherosclerosis in the vascular wall via their systemic effects [9].

Four basic pathogenic mechanisms have been proposed that involve oral inflammations in the pathogenesis of atherosclerosis: (1) low-level bacteremia by which oral bacteria enter the blood stream and invade the arterial wall; (2) systemic inflammation induced by inflammatory mediators, which are released from the sites of the oral inflammation into the blood stream; (3) autoimmunity to host proteins which results from the host immune response to specific components of oral pathogens; (4) pro-atherogenic effects resulting from specific bacterial toxins that are produced by oral pathogenic bacteria.

In this narrative review, we summarize published experimental evidence related to these four mechanisms and discuss the impact in view of the pathogenesis of atherosclerosis. Since this topic is rather broad, a comprehensive consideration of all relevant publications in this area was beyond the scope of this article. Hence, we focused mainly on recent studies published in peer-reviewed high impact journals and we do not claim the completeness of the cited literature.

## 2. Oral Infections: Periodontitis, Gingivitis and Endodontic Lesions

**Figure 1:**
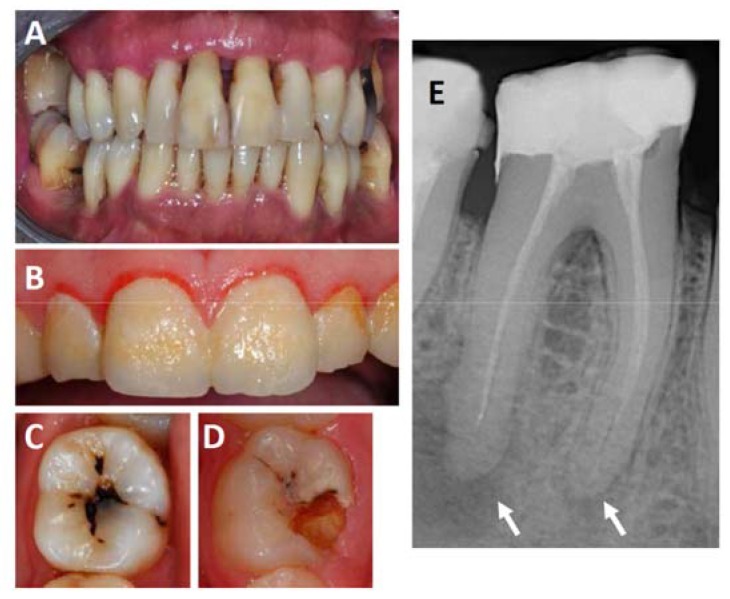
Frequent oral inflammations affecting the teeth, gingiva and the periodontium. (**A**) Shows an example of a severe case of periodontitis. Note the extensive loss of attachment and gingiva recession visible at most teeth; (**B**) shows a case of gingivitis. Note the soft plaque that covers the entire surface of the teeth and the gingiva reddened by the inflammation; panels (**C**,**D**) show examples of teeth affected by root caries, which often lead to the formation of endodontic lesions in the form of periapical abscesses, which can be detected on radiographs as shown in panel (**E**). Arrows mark the locations of periapical abscesses.

Oral infections occur frequently in humans and often lead to chronic inflammations affecting the teeth (caries), the gingival tissues surrounding the teeth (gingivitis and endodontic lesions), and the tooth-supporting structures (periodontitis) (see Figure 1 for example) [10]. Bacteria populating the tooth surface in form of a biofilm can infect the gingiva, which may trigger an immune response in gingival tissues. If the infection persists, it can induce an acute inflammatory reaction known as gingivitis, characterized by swelling, redness and bleeding [11]. Gingivitis is a precursor to periodontitis, which develops if the bacteria and the accompanying inflammation migrate apically along the root surface and penetrate into the tooth supporting structures [12]. In Europe, almost 50% of adults aged 30 years or above suffer from some form of periodontitis and over 10% have severe chronic periodontitis [13]. According to data from the World Health Organization, 5–20% of the adult population worldwide is affected by severe periodontitis defined by the presence of periodontal pockets of ≥6 mm [14].

Besides gingivitis and periodontitis, there are other forms of frequent oral inflammations, most notably endodontic inflammations which typically result from deep dental caries penetrating through the root canal to the apex of the teeth’s root where a periapical abscess is formed [15]. There is currently no solid information available concerning the exact prevalence of endodontic lesions in Europe or elsewhere. However, in several Scandinavian studies, the prevalence of such lesions ranged from 30 to 60%, and increased with age [16,17] These results are in line with more current results from Canada which confirmed the high prevalence of endodontic inflammations in root-filled teeth [18]. Thus, it may be assumed that a significant fraction of most populations is exposed to endodontic inflammations.

## 3. Potential Role of Bacteremia

Pathogenic bacteria originating from the inflamed periodontium may penetrate into the body via the vascular system, either by entering the blood or lymph directly or as internalized particles of immune cells [19,20]. Bacteria, which are early inoculated into the child’s oral cavity by the parents, grow out to create a complex ecosystem comprising more than 700 different bacterial species, which have co-evolved with the human immune system and which populate all oral hard and soft surfaces [11]. Gingivitis and periodontitis are both bacterially induced diseases with a long-standing chronic character; however, they differ from each other with respect to the composition of the biofilms, pathological processes and the sub-types of the infiltrating immune cells. Epithelial cells, which are the first line of defense against the infiltration of bacteria, create a mechanical and chemical defense barrier by targeting bacteria through the release of antimicrobial peptides hBD-2, hBD-3 and the cathelicidin LL-37 and by attracting the migration of immune cells to the inflamed site by secreting chemoattractans, such as IL-1 or IL-8 [21,22,23]. Monocytes, which are accumulating in the soft tissues, produce additional pro-inflammatory mediators [24], thereby contributing to edematous swelling and a high tendency for bleeding of the gingival and periodontal tissues. This may facilitate the penetration of oral bacteria into the bloodstream. Transient bacteremias could be demonstrated in patients with periodontitis after tooth brushing and following periodontal treatment [19,25,26]. A recent meta-analysis based on 63 studies covering 1791 patients confirmed the presence of 23 oral bacterial species in atherosclerotic plaque samples [27]. Five, *Campylobacter rectus*, *Porphyromonas gingivalis*, *Porphyromonas endodontalis*, *Prevotella intermedia*, and *Prevotella nigrescens* were specific for atherosclerotic plaques, whereas the other 18 could also be detected in non-cardiac tissues. It is interesting to note that the detected bacterial species were not be limited to pathogenic species, such as *Porphyromonas gingivalis*, but also included benign species that are generally associated with dental plaque on tooth surfaces [28].

Bacteria from endodontic lesions, such as *Streptococcus mutans*, were also detected in significant quantities in biopsies obtained from heart valves (40% positive) and atheromas (48% positive) [29]. The corresponding signals were considerably stronger than those of other tested bacterial species, including those related to periodontitis. *Streptococcus mutans* can invade vascular endothelial and smooth muscle cells in vitro and, thus, may be able to trigger endothelial dysfunction which could in turn promote atherosclerosis [30].

## 4. Potential Role of Systemic Inflammation

Oral infections, including gingivitis, periodontitis, and endodontic lesions consistently elevate systemic levels of C-reactive protein (CRP), which is a sensitive biomarker for systemic inflammation. One of the first studies published by Boucher et al. [31] showed higher incidence of positive CRP tests and stronger CRP test reactions in samples from patients with acute and chronic endodontic lesions (alveolar abscesses) than from patients with other forms of oral inflammation. Subsequently, various studies showed that patients with less severe oral infections, such as chronic periodontitis, also have higher serum CRP levels than unaffected subjects [32,33,34,35]. The severity of the infection correlates with the CRP level [36,37], and the CRP response was shown to be pathogen-dependent [37,38].

Oral inflammations increase the circulating levels of many other inflammatory markers and cytokines in addition to CRP (for more details see Table 1) [39,40]. The respective lesions secrete large amounts of the pro-inflammatory mediator interleukin-6 (IL-6), which induces the production of CRP and fibrinogen by the liver, resulting in an acute-phase reaction that has pro-inflammatory and pro-atherogenic effects [33]. These results show that oral inflammations are potent inducers of systemic inflammation which may increase inflammatory activity in existing atherosclerotic lesions, thereby increasing the risk of CVD.

## 5. Potential Role of Autoimmunity

Autoimmune processes play an important role in the pathogenesis of atherosclerosis [41,42,43]. Accelerated atherosclerosis and CVD occurring at a young age have been observed in several autoantibody-associated diseases, such as rheumatoid arthritis, systemic lupus erythematosus and antiphospholipid syndrome [44,45,46]. Among the many self-antigens that have been proposed as potential targets of the self-directed immune responses in atherosclerosis [47], heat shock proteins (HSPs) are of special interest, because auto-reactivity to HSPs also occurs in the periodontium of patients with periodontal disease [48].

**Figure 2:**
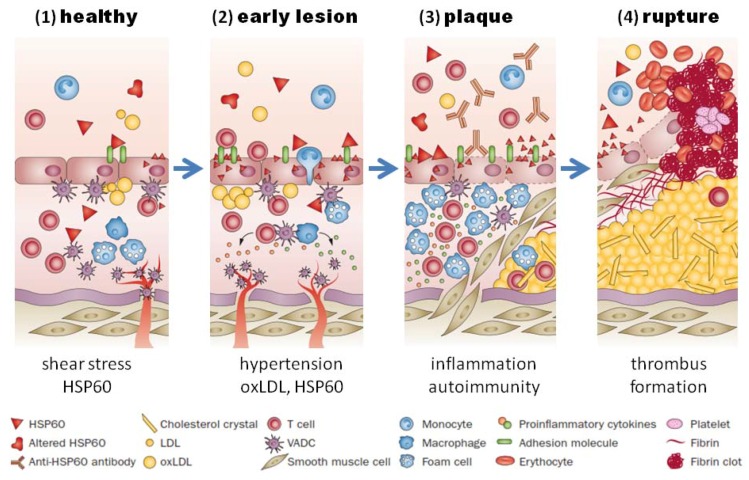
HSP60-induced atherosclerosis. (**1**) Healthy arteries are exposed to hemodynamic turbulences leading to shear stress at curves and branching points, which are prone to atherosclerosis; (**2**) classic risk factors (i.e., high blood pressure) may aggravate the stress response in endothelial cells, which leads to more surface expression of adhesion molecules and HSP60. This, together with secreted HSP60, attracts T cells and other proinflammatory cells to infiltrate the intima. Binding of cross-reactive antibodies to HSP60 to endothelial cells induce an autoimmune response, which promotes endothelial dysfunction and migration of mononuclear cells into the intima; (**3**) plaques start to develop, when macrophages and vascular smooth muscle are transformed to foam cells and produce proinflammatory cytokines. Soluble HSP60 is further released from damaged cells. If the inflammation persists, the lesion becomes more complex and a necrotic core composed of necrotic and apoptotic cells is formed. Cell debris, cholesterol crystals accumulate, and a fibrous cap is formed; (**4**) unstable plaques can rupture which leads to exposure of the core to the blood followed by thrombus formation. Abbreviations: HSP, heat shock protein; oxLDL, oxidized LDL; SMC, smooth-muscle cell; VADC, vascular-associated dendritic cell. Adapted from Servier Medical Art. creative commons license http://creativecommons.org/licenses/by/3.0/legalcode ©Servier.

Heat shock proteins belong to a highly conserved family of molecular chaperones involved in stress protection [49]. *Porphyromonas gingivalis* and many other bacteria involved in oral infections, contain homologs to human HSPs [50]. The HSP60 homolog of *Porphyromonas gingivalis*, which is called GroEL [51], can induce a humoral and cellular immune response in humans. Elevated levels of antibodies and T cells directed against GroEL cross-reacting with HSP60 could be demonstrated in atherosclerotic plaques and periodontal lesions and also in sera from patients with atherosclerosis and periodontitis [52,53,54,55]. The mechanism of HSP60-induced atherosclerosis is schematically illustrated in Figure 2.

Autoantibodies to citrullinated proteins represent another autoimmune mechanism potentially involved in the pathogenesis of atherosclerosis [56]. Protein citrullination is a post-translational modification by which l-arginine is enzymatically converted to l-citrulline by an enzyme called peptidylarginine deiminase (PAD) [57,58]. Humans have five PAD isoenzymes, which fulfill important physiological roles during inflammation, apoptosis, embryonic development and epigenetic gene regulation [58,59]. Healthy humans are generally immune tolerant to citrullinated proteins. However, *Porphyromonas gingivalis*, one of the pathogens causing periodontitis, expresses a prokaryotic PAD enzyme, which citrullinates not only some of its own bacterial proteins but also a number of host proteins (i.e., α-enolase, fibrinogen and vimentin) [60]. It was hypothesized that the long-lasting exposure to highly citrullinated bacterial and host proteins during periodontitis may trigger breakdown of immune tolerance to citrullinated epitopes in susceptible individuals, thereby favoring autoimmunity and the development of rheumatoid arthritis and atherosclerosis [59,61,62,63]. In line with this hypothesis, periodontitis was independently associated with rheumatoid arthritis in multiple epidemiological studies (reviewed in ref. [64]) and a specific set of anti-citrullinated peptide antibodies (ACPA), which was present in rheumatoid arthritis patients, was associated with coronary artery calcification, a well established surrogate marker of coronary atherosclerosis [65].

## 6. Potential Role of Bacterial Toxins

Patients with chronic oral inflammations are exposed to a complex mixture of bacterial components. Lipopolysaccharide (LPS) is an endotoxin produced by Gram-negative bacteria, which has important pro-inflammatory effects [66]. It could be demonstrated that serum LPS level is independently associated with the risk of future CVD [67] and several of its risk factors [68]. *Aggregatibacter actinomycetemcomitans* (a Gram-negative, facultative anaerobe bacterium associated with localized aggressive periodontitis) was recently shown to secrete 179 proteins, including cytolethal distending toxin, leukotoxin A (LtxA) and macrophage infectivity protein [69]. Leukotxin A kills white blood cells by inducing cofilin dephosphorylation and actin depolymerization [70]. When added to human brain endothelial cells in vitro, LtxA led to apoptosis and G2/M phase cell cycle arrest and induced the expression of ICAM-1 and VCAM-1 [71]. In addition, LtxA from *Aggregatibacter actinomycetemcomitans* can induce hypercitrullination of a large number of proteins in host neutrophils [72]. The pore-forming toxin triggers dysregulated activation of host PADs and export of the hypercitrullinated proteins from neutrophils, which may act as citrullinated autoantigenes, favoring the formation of ACPA, rheumatoid arthritis and atherosclerosis. Various strains of *Porphyromonas gingivalis* were shown to secrete up to 200 proteins, including gingipains, agglutination proteins, *P. gingivalis* PAD, and receptor antigens [73]. Rgp and Kgp gingipains were shown to induce lipid peroxidation and to modify human low density lipoproteins (LDL) and high density lipoproteins (HDL) [74].

## 7. Discussion

The presented findings support the argument that chronic oral inflammations likely affect multiple pathways involved in atherosclerosis, and that all four basic mechanisms that were proposed in this context are important. None of these mechanisms is specific for oral inflammations. However, because of their high prevalence and chronic nature, it cannot be excluded at this stage that these inflammations have a profound population-based impact on the atherosclerosis-related disease burden.

The well documented enrichment of oral bacteria or their DNA in atherogenic lesions likely has profound implications. Bacteria and their DNA trigger the innate immune system by activating pattern-recognition receptors (PRRs), such as Toll-like receptors, or TLRs, and NOD proteins, which recognize so-called “pathogen-associated molecular patterns (PAMPs)” and activate multiple pro-inflammatory signaling pathways [75]. In addition to the TLRs, members of the scavenger receptor family are involved in microbial pattern recognition [76]. The scavenger receptors SR-A and CD36 mediate down-regulation of macrophage activation and contribute to the phagocytosis of apoptotic cells [77]. Besides recognizing PAMPs, these receptors are also involved in the uptake of oxidized LDL by macrophages, which play a causative role in the pathogenesis of atherosclerosis [78].

The well-documented induction of systemic inflammation by chronic oral inflammations implies that the affected individuals are at increased risk of CVD. It could be demonstrated that elevated concentrations of CRP, IL-6 and fibrinogen are associated with increased 10-year risk of CVD [79,80]. Recent data from the randomized controlled CANTOS trial showed that anti-inflammatory treatment with canakinumab reduced the rate of recurrent cardiovascular events in patients with CVD compared to placebo [81]. Canakinumab is a human monoclonal antibody that neutralizes IL-1β, an inflammatory protein that is elevated in states of systemic inflammation [82] and during periodontitis [83]. Blocking IL-1β resulted in reduced progression of periodontal bone loss and attachment loss in a non-human primate model of periodontitis [84,85].

Activation of the HSP60 autoimmunity mechanism has been firmly established to operate in patients with periodontitis infected with *Porphyromonas gingivalis*. Humoral and cellular immunity against HSP60 is a normal feature of healthy humans, which participates in the protection against microbial infections [86]. If endothelial cells are subjected to stressful conditions, such as hemodynamic stress, they over-express HSP60, which is then also presented on the cells’ surface, and the cross-reactive anti-HSP60 antibodies may damage the endothelial cells, thereby initiating a repair response which triggers endothelial dysfunction and the subsequent development of atherosclerosis (Figure 2) [87]. Since the ability of the immune system to generate this potentially dangerous immune response depends not only on the strength of the HSP60 immuno response, which is elevated in patients with periodontitis, but also on the highly polymorphic MHC class I and II epitopes, that are expressed on the cells’ surface, individuals differ with respect to their susceptibility to HSP60-induced atherosclerosis [88].

Whether the HSPs of endodontic pathogens elicit a similar immune response as was demonstrated for *Porphyromonas gingivalis* GroEL, has so far not been addressed. Since many studies have shown an association between CVD and endodontic lesions [89,90,91,92,93,94,95], which is of similar strength compared to the association between CVD and periodontitis, it would be of interest to confirm that the mechanism is also operating in these patients.

Citrullinated proteins, especially fibrinogen, were identified in atherosclerotic plaques [56], suggesting that the atherosclerotic vessel wall might be a target for ACPA mediated autoimmunity induced by periodontal inflammations. Although the prevalence of ACPA positivity is high in rheumatoid arthritis patients (>20%), it is only ~1% in the general population [96]. Thus, this mechanism may be relevant mainly in patients with rheumatoid arthritis.

Profound technological advances in areas such as genomics, proteomics and metabolomics have recently led to the identification of a vast number of factors that are secreted by oral pathogens (“secretome”), some of which might influence the host immune system and the pathogenesis of atherosclerosis. These factors warrant further characterization with respect to their specific effects. This fascinating area of research has a high potential to identify factors that may lead to the development of drugs.

In a scientific statement published in 2012, the American Heart Association concluded that “observational studies supported an association between periodontitis and atherosclerosis independent of known confounders” [97]. However, because it was unclear whether periodontal interventions could prevent atherosclerosis or modify its outcomes in the long-term, the evidence did not support a causative relationship. Despite all the recent progress regarding insights into the potentially involved mechanisms, this limitation still exists.

## Figures and Tables

**Table 1:** Cytokines acting in atherosclerosis and oral inflammations.

Cytokine	Familiy
IL-8, MIP-1, MCP-1, RANTES	Chemotactic
IL-1α, IL-1β, TNFα, IL-6, PAF	Pro-inflammatory
IL-1RA, IL-4, IL-10	Anti-inflammatory
IFN-γ, IL-2, IL-4, IL-5, IL-7	Immunoregulatory
PDGF, EGF, FGF, IGF, VEGF	Growth factor

EGF, epidermal growth factor; FGF, fibroblast growth factor; IFN, interferon; IGF insulin-like growth factor; IL, interleukin; IL-1RA, interleukin-1-receptor antagonist; MIP, macrophage inflammatory protein; MCP, monocyte chemotactic protein; PAF, platelet activating factor; PDGF, platelet derived growth factor; RANTES, regulated upon activation, normal T cell expressed and secreted; VEGF, vascular endothelial growth factor.
